# Experiences of maltreatment in childhood and attention to facial emotions in healthy young women

**DOI:** 10.1038/s41598-022-08290-1

**Published:** 2022-03-12

**Authors:** Dennis Hoepfel, Vivien Günther, Anna Bujanow, Anette Kersting, Charlott Maria Bodenschatz, Thomas Suslow

**Affiliations:** Department of Psychosomatic Medicine and Psychotherapy, University of Leipzig Medical Center, Semmelweisstr. 10, 04103 Leipzig, Germany

**Keywords:** Psychology, Risk factors

## Abstract

Using reaction-time measures, research on the relationship between childhood maltreatment and biased attention to emotional stimuli in adults has obtained inconsistent results. To help clarify this issue, we conducted an eye-tracking study on the link between childhood maltreatment and allocation of attention to facial emotions analyzing gaze behavior in addition to manual reactions. In contrast to prior investigations, we excluded individuals with tendencies to minimize maltreatment experiences from analyses. Gaze behavior and manual response time of 58 healthy women were examined in a dot-probe task in which pairs of emotional (happy, sad, or disgusted) and neutral faces were presented. In our analyses, participants’ affectivity, level of alexithymia, and intelligence were controlled. Entry time and dwell time on facial expressions were used as indicators of attention allocation. Childhood maltreatment showed no effect on response latencies but was associated with shorter entry times on emotional faces and shorter dwell time on disgusted faces. Experiences of childhood maltreatment seem to be linked to an increased early vigilance to emotional social signals and to an attentional avoidance of hostile facial expressions at a later stage of perception. The present results suggest a vigilance-avoidance pattern of attention allocation associated with childhood maltreatment.

## Introduction

Child maltreatment can take various forms. It can include acts of commission like sexual, physical, and emotional abuse and acts of omission like physical and emotional neglect by parents or other caregivers that result in harm or threat of harm to a child^1^. Experiences of maltreatment during childhood are widespread. In high-income countries, up to 16% of children suffer physical abuse and about 10% are neglected or psychologically abused every year^2^. The consequences of maltreatment can be long-lasting and have a sustained negative impact on mental and physical health^3,4^. Childhood traumata are a strong risk factor for developing mental disorders^5^. However, the exact mechanisms through which maltreatment increases the vulnerability to emotional disorders such as depression and anxiety disorders have not been clarified.

Important factors that have been proposed to contribute to the development of emotional dysfunctions are biases in attention to threatening and other negative stimuli^6^. Attentional biases are tendencies to have one’s attention initially drawn and sustained by a specific category of stimuli over another. Habitual biases toward specific types of emotional stimuli can be considered a rapid, unconscious form of attention deployment^7^. Fast identification and increased processing of threatening cues has evolutionary advantages as it helps to prepare defensive responses and reduces the risk of being harmed^8^. Repeated experiences of violence and stress in early phases of neurocognitive development when children are strongly dependent on caregivers seem to lead to a heightened sensitivity to negative social information^9^ and to have a profound adverse impact on neural pathways that underly emotion perception and regulation^10^. One of the most frequently used experimental tasks for assessing attention biases is the dot-probe task^11^. In this task, two stimuli, one neutral and one emotional, appear randomly on different sides of a computer screen, before a dot is presented in the location of one stimulus. Participants should indicate the location of the dot as quickly as possible. Faster reaction time to the dot when it occurs in the previous location of an emotional stimulus is interpreted as vigilance to emotional information^12^.

Using the dot-probe paradigm, research on the relationship between childhood maltreatment and biased attention to emotional stimuli has obtained contradicting results. There are studies showing a positive association between experiences of childhood maltreatment and a bias to orient attention towards angry or negative faces^13,14^. Other studies observed that maltreated children and adults with a history of maltreatment demonstrate tendencies to direct attention away from threatening or negative stimuli^15,16^. Maltreated subjects may have learned to allocate their attention away from potential stressors as a means of coping with persistent or uncontrollable adversity. Further studies found no evidence for relations of childhood maltreatment with attentional biases either toward or away from threatening or negative cues^17,18^. These mixed findings could be due to the poor psychometric properties of the dot-probe task. Studies on the reliability of the dot-probe paradigm have yielded low internal and retest coefficients^19,20^. Against this background, attentional bias scores derived from the task should be considered with caution. Indirect measures of attention biases based on response latencies appear to have methodological weaknesses which might be overcome with more direct measures of attention such as the examination of participants’ gaze behavior during the dot-probe task.

In the last decades, eye-tracking technology has become increasingly important in clinical research to better understand biased attention in the perception of emotional stimuli^21,22^. Eye-tracking provides a rather direct measure of attention allocation, as the focus of attention and the direction of the gaze are assumed to be coupled^23^. It allows a spatially and temporally more detailed assessment of attention allocation compared to measures that are based on manual reaction times. Reaction latencies provide only single data points per trial with which it is impossible to make precise inferences about the sequential operations involved in the deployment of attention^24^. Through the registration of eye movements during the dot-probe task, it is possible to analyze, for example, how fast a stimulus in a pair was fixated or how long each of the two displayed stimuli was looked at.

In the present eye-tracking study, we examined the association of childhood maltreatment with allocation of attention to facial emotions in a dot-probe task. The present work should help to clarify whether experiences of childhood maltreatment go along with attentional biases toward or away from emotional and, in particular, negative social stimuli.

There is evidence for a female advantage in recognizing facially expressed emotions^25,26^ but also for gender differences in attentional biases toward emotional faces^27^. Furthermore, there appears to exist gender differences in the exposure and reactions to adverse childhood experiences which might be at least partially due to genetic difference ^28,29^ and which could contribute to the differential risk for pathogenesis of mental health disorders among adults^30^ Against this background and since men and women also differ in their neural response in the dot-probe task while allocating attention to emotional facial expressions^31^, we only included women in our investigation to examine a gender homogeneous sample. To our knowledge, no prior investigation on childhood maltreatment using the dot-probe task has explored its relationships with attentional biases analyzing gaze behavior in addition to manual reaction times. In our experiment, we showed an emotional (happy, sad, and disgust) combined with a neutral facial expression for 1 s before the dot appeared. Thus, we presented one positive (happiness) and two negative (sadness and disgust) emotion qualities. The expressions of happiness and sadness signal an invitation for social interaction and approach of some sort. Happy facial expressions are invitations to the perceiver to approach the expresser, whereas sadness is a signal that the expresser feels helpless and needs care and support^32^. Instead, disgust is a hostility- and threat-related emotion. The facial expression of disgust signals revulsion, social disapproval, and interpersonal rejection^33^.

Our analysis of eye-movements is focused on two parameters: the initial orienting of gaze to the emotional face (i.e., the entry times on the emotional and the neutral face) and the duration of attention allocation to the emotional versus the neutral face (i.e., the time participants’ gaze remains fixated on the emotional and the neutral face). Based on the findings from previous eye-tracking research^34^ we expected that the entry times to emotional faces are shorter, and the dwell times on emotional faces are longer than those for neutral expressions in the total sample. Moreover, we hypothesized that the analysis of manual reaction times would indicate vigilance for emotional faces. That is, reaction times to the dot were expected to be faster when it occurred in the previous location of an emotional compared to a neutral stimulus. As the findings of previous studies on childhood maltreatment and attention to emotional stimuli are inconsistent, we stated non-directional hypotheses and assumed that childhood maltreatment would be related to attention allocation to emotional faces, both as measured by reaction times and as assessed by eye-tracking parameters.

To assess childhood maltreatment, we administered the Childhood Trauma Questionnaire (CTQ; ^35,36^) in our study. The CTQ is an internationally widely used instrument for retrospectively recording early-life traumatization. Retrospective reports of childhood maltreatment have been criticized for ignoring the tendency in some individuals to underreport trauma experiences compared to other methods of assessment, e.g., sibling interviews^37^. The CTQ scale’s authors were aware of the problem that some people trivialize or deny experiences of maltreatment and therefore included a response bias subscale, the Minimization-denial scale. Even though minimization appears to be rather common (about 40% minimize in healthy samples^37^), in the past, the overwhelming majority of studies that present CTQ data did not report minimization-denial tendencies or took them into account in their analyses^38^. Against this background, the effects of early maltreatment as assessed by the CTQ can be underestimated. In order to avoid this problem, we identified study participants with positive MD scores and excluded them from data analysis.

State and trait affectivity as well as depression and anxiety of study participants were assessed and controlled in our study because visual attention has shown to be biased towards mood-congruent stimuli^39^. In particular, level of depressive symptoms and anxiety have been found to be associated with heightened attention for threatening or negative stimuli in healthy individuals^40,41^. As the personality trait alexithymia is known to have an adverse impact on emotion processing^42^ we decided to also measure and control this personality characteristic in our investigation.

## Results

### Relations of childhood maltreatment with positive and negative affectivity, alexithymia, and intelligence

Table 1 summarizes the correlations between CTQ and all control variables used in the regression models. The analysis yielded no significant correlations between traumatic childhood experiences and any positive or negative affect scale (as assessed by the PANAS). There was a small to moderate positive correlation of traumatic childhood experiences with level of depressive symptoms. No correlations of the CTQ were observed with state and trait anxiety, intelligence, and alexithymia (see Table 1 for details).

**Table 1:** Descriptive statistics of and correlations between self-report and test scores (N = 58) with Cronbach’s alpha for self-report questionnaires.

Variable	1	2	3	4	5	6	7	8	9	α
1. CTQ										.88
2. PANAS-PA S	− .19									.88
3. PANAS-NA S	− .03	− .17								.81
4. PANAS-PA T	− .05	.62**	− .25							.85
5. PANAS-NA T	.07	− .27*	.71**	− .14						.83
6. STAI S	.19	− .60**	.54**	− .70**	.40**					.91
7. STAI T	.10	− .37**	.62**	− .53**	.65**	.70**				.89
8. BDI-II	.28*	− .19	.45**	− .45**	.28*	.44**	.60**			.77
9. TAS-20	.14	− .20	.43**	− .36**	.55**	.47**	.72**	.49**		.84
10. MWT-B	− .07	.04	− .03	.07	− .05	.06	− .11	− .01	− .23	–
	1	2	3	4	5	6	7	8	9	10
Mean	37.34	3.20	1.32	3.36	1.59	36.05	39.09	7.47	44.02	31.05
SD	10.60	0.61	0.34	0.59	0.46	8.43	8.56	4.83	11.05	2.56

*CTQ* Childhood Trauma Questionnaire, *PANAS-PA S* Positive and Negative Affect Schedule, positive affect scale, state (mean item score), *PANAS-NA S* Positive and Negative Affect Schedule, negative affect scale, state (mean item score), *PANAS-PA T* Positive and Negative Affect Schedule, positive affect scale, trait (mean item score), *PANAS-NA T* Positive and Negative Affect Schedule, negative affect scale, trait (mean item score), *STAI S* State Trait Anxiety Inventory, state version, *STAI T* State Trait Anxiety Inventory, trait version, *BDI-II* Beck Depression Inventory, *TAS-20* 20-Item Toronto Alexithymia Scale, *MWT-B* Mehrfachwahl-Wortschatz-Intelligenztest version B.

*p < .05; **p < .01.

### Dot-probe task

#### Response times and relations with childhood maltreatment

Trials with missing (0.30%) or invalid (0.03%) responses in the dot-probe task and extreme outliers with response times lower than 100 ms and higher than 3000 ms (1.83%) were excluded from the analyses. We also discarded trials with response times lower or higher than 2 standard deviations from the subject’s mean response time of the corresponding emotion as individual outliers (3.85%). Altogether, we excluded 6% of all trials from our analysis.

**Figure 1:**
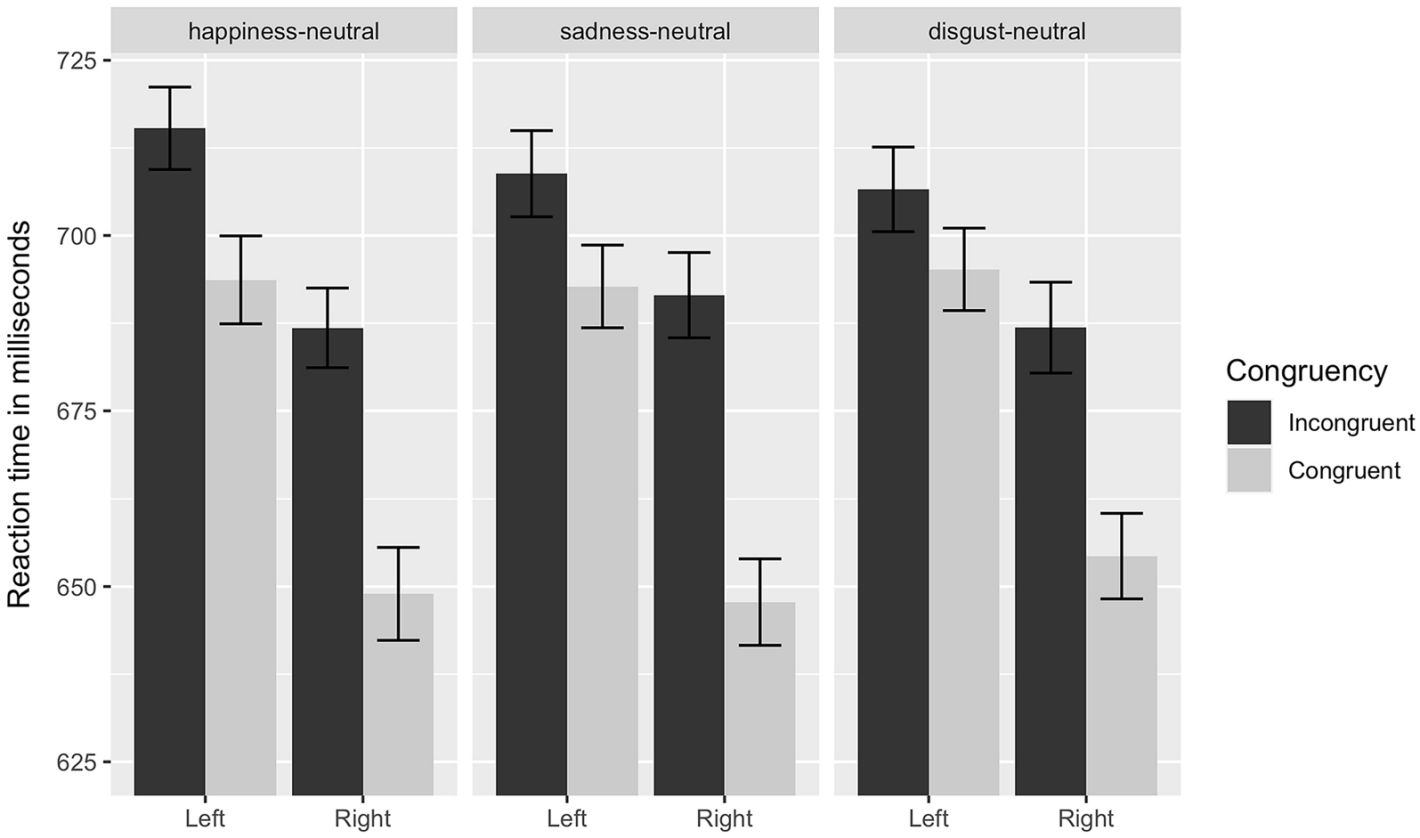
Reaction times in ms for happy-, sad-, and disgusted-neutral facial expression pairs as a function of congruency and location (i.e., (bottom) left, or (top) right position of the emotional facial expression). Error bars denote standard error.

Figure 1 displays the mean reaction latencies as a function of location, congruency, and facial expression pair condition. The analysis of mean reaction times yielded significant main effects of location, *F(1, 633)* = 95.25, *p* < 0.001, ${\upeta }_{P}^{2}$ =*0.13,* with shorter reaction times to probes that were displayed on the top-right position, and congruency, *F(*1, 633) = *67.85, p* < 0.01, ${\upeta }_{P}^{2}$ = *0.10,* with shorter reaction times to dots that were displayed at the position of the emotional face, but no main effect of facial expression pair, *F*(2, 633) = *0.01, p* = 0.99*.* Thus, it can be noted that response times to the dot were faster when it occurred in the previous position of the emotional face suggesting vigilance to emotional expression regardless of the emotional quality. The interaction between congruency and position was significant, *F*(1, 633) = 9.48, *p* < 0.05, ${\eta }_{P}^{2}$ = 0.01, indicating that the difference in mean reaction times between incongruent and congruent trials was larger for probes that were displayed at the top right position (see Fig. 1). Childhood maltreatment as assessed by the CTQ showed no significant main or interaction effect on response times.

#### Dwell times and relations with childhood maltreatment

**Figure 2:**
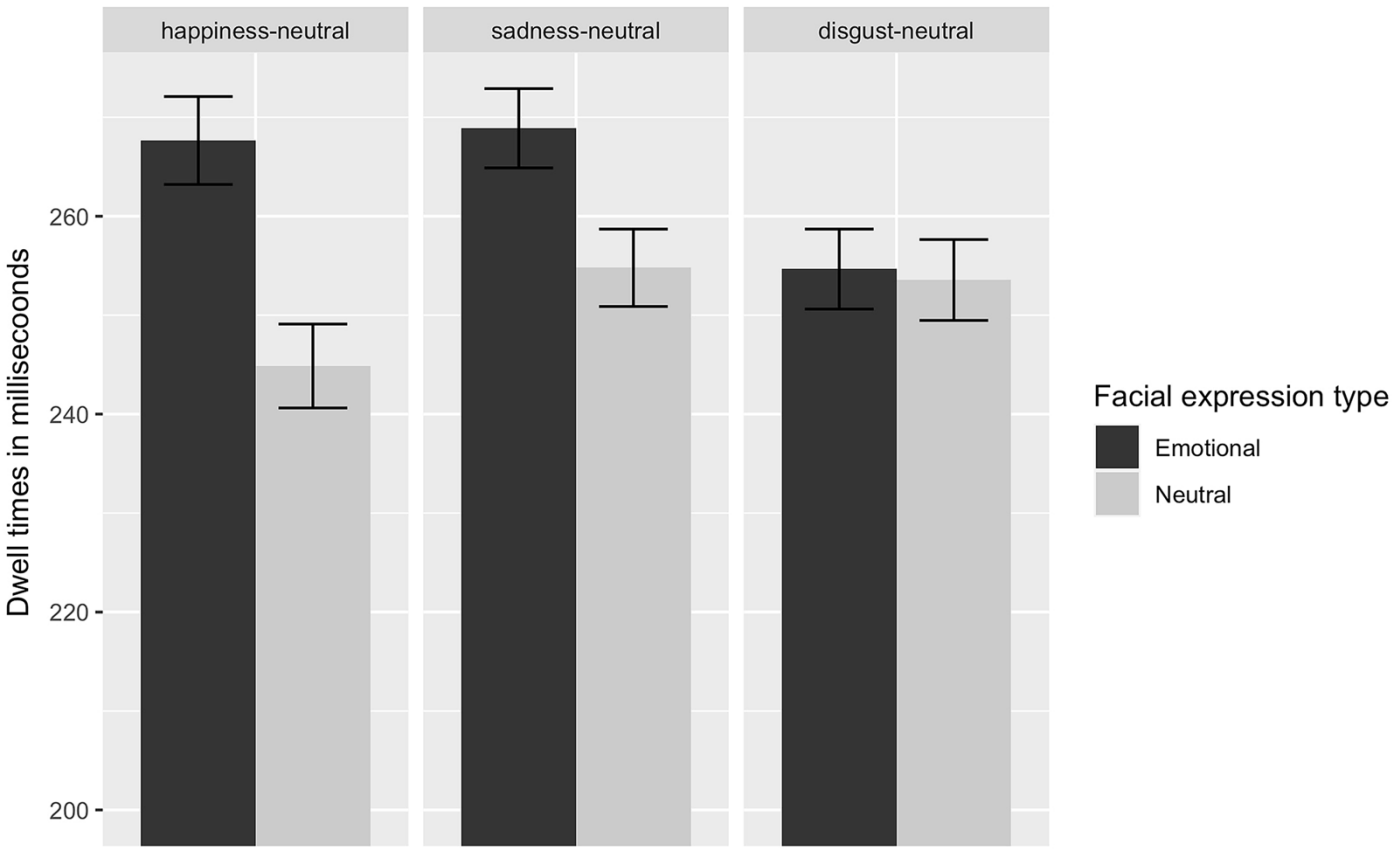
Dwell times on the emotional and neutral facial expressions in ms for happy-, sad-, and disgusted-neutral facial expression pairs. Error bars denote standard error.

**Figure 3:**
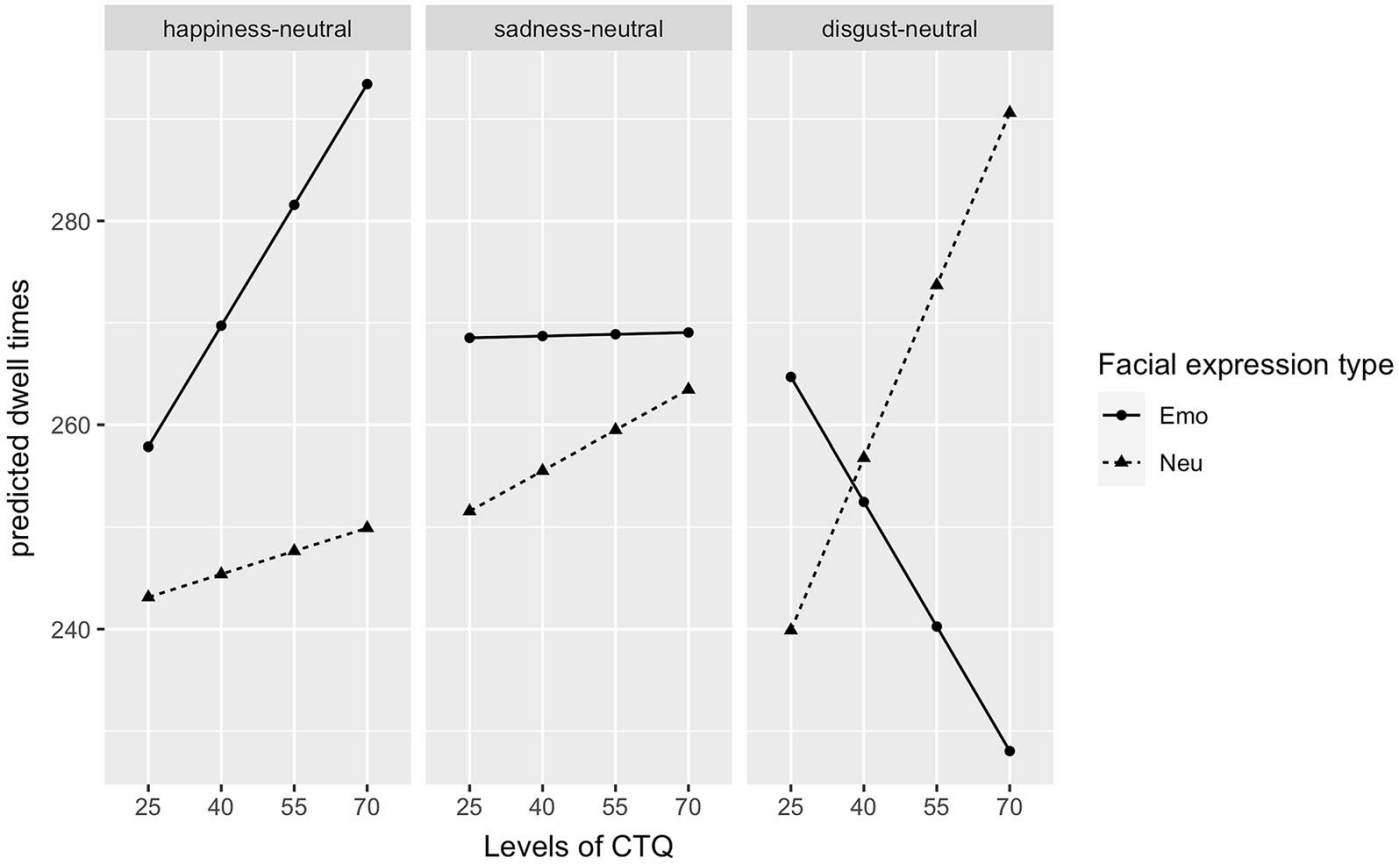
Predicted dwell times on emotional and neutral facial expressions as a function of facial expression pairs and different levels of CTQ.

Mean dwell times for the emotional and neutral stimuli as a function of stimulus pairs are presented in Fig. 2. The analysis of dwell times yielded a significant main effect of facial expression type, *F*(1,280) = 4.41, *p* < 0.05, ${\eta }_{P}^{2}$ = 0.02, with longer dwell times on the emotional facial expressions, but no main effects of facial expression pair, *F(*2,280) = 0.47, *p* = 0.63, and CTQ, *F*(1,45) = 0.04, *p* = 0.84. There were no significant two-way interactions, but a significant three-way interaction of facial expression type, facial expression pair, and CTQ score, *F*(2,280) = 3.76, *p* < 0.05, ${\eta }_{P}^{2}$ = 0.03. To better understand this interaction, we illustrated the predicted dwell times of the facial expression types for each facial expression pair for different levels of CTQ in Fig. 3. Dwell time on the emotional facial expression decreases significantly for the disgust-neutral condition, while dwell time for the neutral facial expression increases significantly with increasing CTQ score (*p*s < 0.05). No such effect is found for the happiness-neutral, *t* (280) = 0.95 *p* = 0.34, and sadness-neutral conditions, *t* (280) =−0.37, *p* = 0.71.

#### Entry times and relations with childhood maltreatment

**Figure 4:**
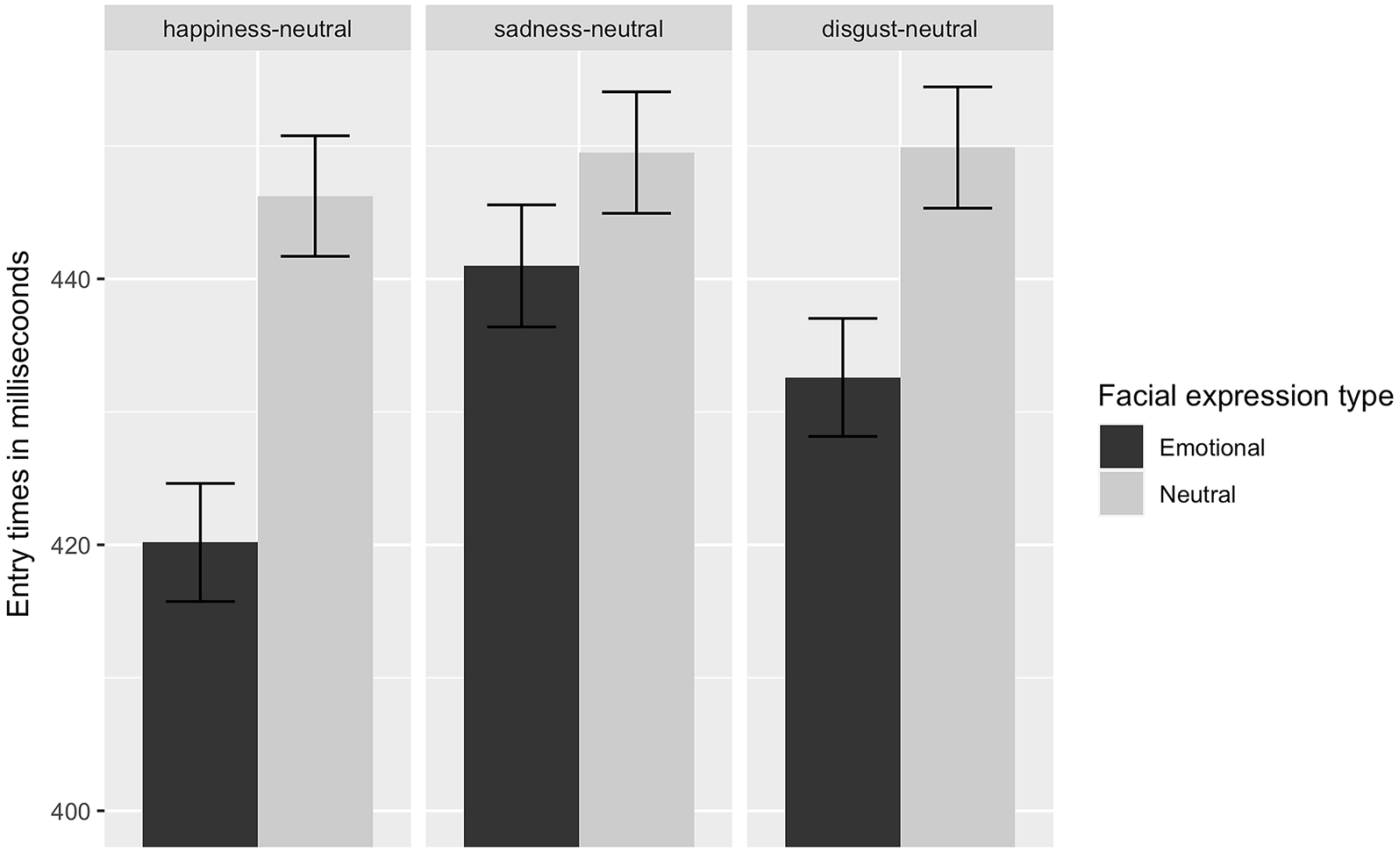
Entry times on the emotional and neutral facial expressions in ms for happy-, sad-, and disgusted-neutral facial expression pairs. Error bars denote standard error.

Figure 4 presents the mean entry times for emotional and neutral facial expressions for each facial expression pair.

**Figure 5:**
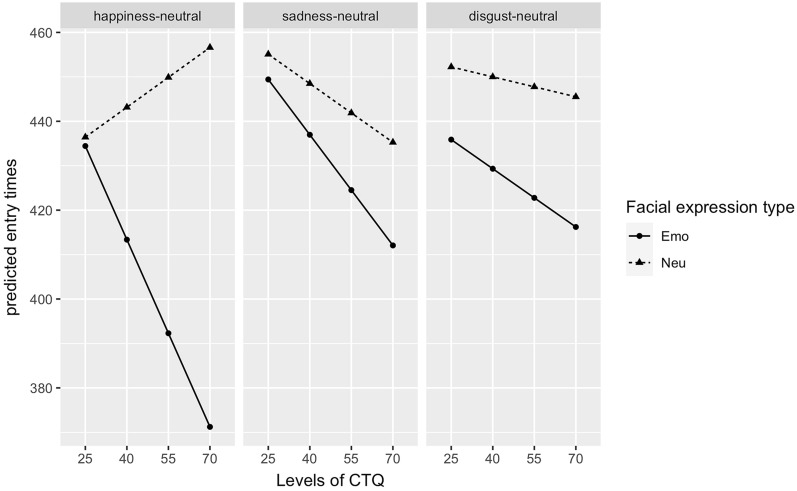
Predicted entry times on emotional and neutral facial expressions as a function of facial expression pairs and different levels of CTQ.

The model revealed no main effects of facial expression type, *F*(1,280) = 0.64, *p* = 0.42, facial expression pair, *F*(2,280) = 0.60, *p* = 0.55, or CTQ score, *F*(1,45) = 1.34, *p* = 0.24, but a significant two-way interaction between facial expression type and CTQ, *F*(1,280) = 3.97, *p* < 0.05, ${\eta }_{P}^{2}$ = 0.01. Figure 5 illustrates this interaction effect and shows that high CTQ scores were related to significantly shorter entry times on the emotional, but not the neutral facial expressions (*p* < 0.05).

#### Relations between response times and eye-tracking parameters

Table 2 presents the results of our correlation analyses for response-, dwell- and entry times. No significant correlation of response times with the eye-tracking parameters, i.e., dwell and entry time, was revealed (neither for emotional nor for neutral facial expressions). We found significant correlations of medium effect size between dwell and entry time on emotional and neutral facial expressions (see Table 2 for details). Dwell times on emotional and neutral facial expressions were negatively correlated, as well as entry times on emotional and neutral facial expressions. These correlations indicate that long dwell times and low entry times on one type of facial expression are linked to short dwell times and high entry times on the other type of expression. We also found significant negative correlations between dwell and entry time on emotional facial expressions, as well as dwell and entry time on neutral facial expressions, indicating, that subjects had the tendency to dwell longer on facial expressions they entered early and dwell shorter on facial expressions they entered late. Lastly, we observed small positive correlations between dwell time on emotional and entry time on neutral facial expressions, and dwell time on neutral and entry time on emotional facial expressions, revealing relationships between short dwell times on one type of facial expression and short entry times on the other expression, and vice versa (see Table 2).

**Table 2:** Correlations between response time, dwell time and entry time.

Variable	1	2	3	4
1. Response time				
2. Dwell time emotional	.00			
3. Dwell time neutral	.00	− .53*		
4. Entry time emotional	− .01	− .35*	.25*	
5. Entry time neutral	.00	.26*	− .35*	− .79*

*p < .01.

## Discussion

Experiences of childhood maltreatment are common in the general population^2,43^ and have adverse effects on mental and physical health which often persist into adulthood^3^. Childhood traumata are a strong risk factor for developing anxiety and depressive disorders^44^. One mechanism through which maltreatment may increase the vulnerability to emotional disorders are biases in attention to threatening and other types of negative information^6^. In the present eye-tracking study, we investigated the relation of childhood maltreatment with allocation of attention to facial emotions in a dot-probe task, a widely used experimental paradigm to assess attention biases^11^. The present analysis of gaze behavior and manual response times can help to clarify whether experiences of childhood traumata go along with attentional biases toward or away from emotional stimuli. In our examination of the relationships between childhood maltreatment and attention to facial emotions we controlled several relevant variables, i.e., positive, and negative state and trait affect, state and trait anxiety, depressive symptoms, alexithymia, and intelligence. Prior reaction-time based research on the relationship between childhood maltreatment and biased attention to emotional stimuli has obtained contradicting results: some studies revealed associations of childhood maltreatment with an attention bias towards angry or negative faces^13,14^, other studies reported associations with tendencies to direct attention away from threatening or negative stimuli^15,16^ or found no correlations at all between childhood maltreatment and attention biases^17,18^. Unlike in our study, previous dot-probe research did not control tendencies in study participants to minimize or deny maltreatment experiences.

Our examination of eye-movements was focused on entry time to emotional and neutral faces, an index of initial attention orienting, and dwell time on emotional and neutral faces, an index of sustained attention. The analysis of our entry time data only indicated a two-way interaction between CTQ and facial expression type. Experiences of childhood maltreatment were linked to shorter entry times on the emotional compared to the neutral facial expressions. This means that more experiences of childhood traumata went along with a faster orienting toward emotional expressions independent of emotional quality. It appears that childhood maltreatment could have led to a generally heightened early vigilance to emotional social signals.

The analysis of dwell times showed first that participants’ gaze remained longer on emotional compared to neutral facial expressions. Happy, disgusted, and sad faces attracted attention to a similar extent. This observation is consistent with findings from previous psychological and eye-tracking research^34,45^ suggesting that attention is captured by the affective content of stimuli with positive or negative valence, when they compete for attention with simultaneously presented neutral pictures. Second, the results of our linear model indicated a three-way interaction of CTQ, facial expression type, and facial expression pair. Dwell time on disgusted facial expression diminished with increasing experiences of childhood maltreatment. Such an effect was not found for happy and sad faces. Thus, we observed evidence that individuals with more experiences of childhood traumata tend to allocate their attention less to hostile facial expression compared to those with few or no experiences of childhood traumata. Duration of attention to sad and positive facial expression was not modulated by childhood maltreatment. The present dwell time data are partially in accordance with findings from a previous eye-tracking study based on a 10 s free-viewing paradigm which indicated that depressed patients with a history of childhood maltreatment avoided processing of angry facial expressions^46^.

According to the results from our analysis of response times, decision latencies were faster when the dot occurred in the previous position of the emotional face compared to when it occurred in the position of the neutral face. The present data indicate a vigilance to emotional compared to neutral facial expression regardless of the emotional quality. Study participants allocated more attention to happy, disgusted, and sad faces than to neutral faces. These results are consistent with previous research showing that in general emotional facial expressions accelerate attentional engagement and delay attentional disengagement due to their communicative significance^47^. From an evolutionary perspective it can be argued that emotional facial expressions are prioritized during resource-limited information processing because of their adaptive significance^48^. Importantly, childhood maltreatment showed no effect on response latencies. This means that experiences of childhood traumata were found to be unrelated to attention allocation as assessed by response times. In the past, the dot-probe paradigm has been repeatedly criticized as unreliable and lacking validity^19,20^. However, it must be emphasized that for our dot-probe task a main effect of facial emotion on attention was obtained which supports the validity of the task.

In our study, we observed no correlations between eye-tracking parameters and response times, i.e., direct, and indirect measures of attention allocation. Thus, dwell and entry times as assessed by gaze behavior were found to be independent from manual decision latencies. Our results are largely consistent with findings from previous bias research with the dot-probe task indicating very few correlations between response time and eye-movement parameters^49^. Similarly, no associations were reported between response time and electrocortical indices of attention allocation in an event-related potential study based on the dot-probe task^50^. A possible explanation for this independence of parameters is that entry time is a direct measure of initial attention orientation to the onset of an emotional image and dwell-time a direct measure of late attention allocation to and maintenance on the emotional image, whereas the behavioral measure reflects the response to a separate target stimulus (rhombus or circle) that is presented *after* the presentation of the emotional-neutral facial stimulus pair. Manual motor responses were given on average about 650 to 700 ms after disappearance of the facial stimulus pairs (and 1650 to 1700 ms after the onset of facial stimuli). This means, that the response time measure comprises processes which temporally succeed the attentional processes assessed by the eye-tracking parameters and it includes perception and identification of a geometric symbol and a manual motor response on a computer keyboard which are not part of our eye-tracking measures. Hence there exist substantial differences between response time and eye-tracking parameters of attention regarding procedural and temporal characteristics of their measurement construct.

Another explanation for the observed independence of response time and eye-tracking indices of attentional biases could be that the processes measured by response time do not rely on direct attention allocation. Reaction-time based measures could assess primarily *covert* processes of attention orientation in the dot-probe task. Covert visual attention relates to mental shifts of attention without physically directing the eyes to a stimulus. Covert attention can be deployed to multiple locations simultaneously and is useful to monitor visual scenes^51^. There is evidence from dot-probe research that the reaction-time bias is not linked to the overt orienting of gaze^52^. It even seems that reaction-time biases are more easily detectable when eye movements are absent or restricted^53^.

The results of the current study should be considered in the light of several limitations. History of childhood maltreatment was retrospectively assessed by the CTQ. The cross-sectional design is also a limitation of our study as it does not allow drawing causal conclusions. The generalizability of our results is limited by the fact that we only included well-educated healthy young women who do not represent the general population. This is a substantial limitation of our study. Future research is necessary to clarify the association of maltreatment experiences in childhood with attention to facial emotions in men. This is all the more necessary in view of the sex differences in facial emotion recognition^26^, in attention allocation to facial emotions ^27^, and in exposure as well as reactions to adverse childhood experiences^28,29^. The present results also have to be interpreted with due caution because of a right-skewed distribution of our CTQ data and a rather low number of individuals (n = 14) with elevated CTQ scores (> 40) in our sample. To avoid this problem, we suggest conducting comparisons of extreme groups (e.g., individuals with no experiences of childhood maltreatment vs. individuals with severe experiences of childhood maltreatment) in future studies on the effect of adverse childhood experiences on emotion perception. Another limitation is that static images were used in our study, yet emotional facial expressions in daily life are in general dynamic. In our study, the pairs of facial expressions were displayed for only one second. That means that we investigated attentional processes over a rather short period of time.

As the dot-probe task has shown poor psychometric properties^19,20^ future attentional bias research may consider measuring subjects’ electrocortical activity during the test. Event-related potentials can reveal initial shifts of visual attention to the threatening stimulus in the dot-probe task, as reflected in the N2pc component^50^. In contrast to reaction-time bias scores in the dot-probe task the N2pc was found to be internally reliable^54^.

In sum, our eye-tracking data provide evidence for associations of childhood maltreatment with an increased early vigilance to positive and negative emotional social signals and later to an attentional avoidance of disgusted facial expressions. Disgust is a hostility-related emotion associated with conflict and aggression^55^. The facial expression of disgust communicates social disapproval, and interpersonal rejection^33^. Individuals with a history of childhood maltreatment could be especially afraid of being rejected and disliked by others. Our eye-tracking results can be interpreted within the framework of the vigilance-avoidance theory, a well-established cognitive model in the examination of attention to threat^56,57^. The theory postulates a specific pattern of attention deployment in anxious individuals confronted with threat-related stimuli, characterized by an initial stage of attention allocation to the stimulus, and a subsequent stage of attentional avoidance^58^. Initial vigilance for aversive stimuli promotes early detection of threat and hence enables rapid defensive reactions and reduces risks of being injured or damaged^8^. Avoidance of aversive information in the later phases of attention allocation could have the functions to reduce experiences of anxiety or other negative emotions and to attenuate or evade conflicts. Maltreated individuals may have learned to allocate their attention away from hostile stressors as a means of coping with persistent or uncontrollable adversity. Poor abilities in regulating negative emotions may underlie an avoidant attentional style. There is evidence that early life adversity is linked to deficits in adaptive emotion regulation^59^.

Repeated experiences of violence and neglect in early phases of neurocognitive development when children are strongly dependent on caregivers appear to have profound adverse impacts on neural pathways that underly emotion perception^10^. Our finding of a generally heightened early vigilance to emotional social signals is in line with results from a neuroimaging study showing a general amygdala hyperresponsivity to emotional facial expressions (i.e., to fearful, angry, sad, and happy faces) in adults with a history of childhood maltreatment^60^. Individuals with early experiences of maltreatment may interpret all facial expressions as highly salient. It is possible that the increased vigilance to happy faces reflects a heightened sensitivity toward safety signals from others. However, it can also be argued that individuals with experiences of childhood maltreatment could perceive happy facial expressions as masking malevolent emotions, or they might be interpreted negatively as signs of being ridiculed and laughed at^61^. A generally increased emotional sensitivity may be a factor contributing to difficulties in interpersonal relationships which are frequently observed in adults with a history of childhood maltreatment^62^.

## Materials and methods

### Participants

The final sample consisted of 58 healthy women with a mean age of 24.19 years (*SD* = 3.43, range 18–30). Participants were recruited via public and online notices at the University of Leipzig. All participants were German native speakers. They were screened for multiple exclusion criteria, pregnancy, compromised vision, current or previous mental or neurological illnesses, and abuse of alcohol or other substances before taking part in the study. All subjects gave written informed consent before participating in our investigation.

The study was approved by the ethics committee of the University of Leipzig, Medical School (reference number 239/17-ek) and conducted in accordance with the Declaration of Helsinki^63^. Our initial sample included 91 women. 33 women had a score > 0 on the Minimization-denial scale of the *Childhood Trauma Questionnaire* (CTQ^35,36^) and were excluded from data analysis.

### Self-report questionnaires

#### Childhood maltreatment

We administered the German version of the *Childhood Trauma Questionnaire* (CTQ^36^) to assess participants’ traumatic experiences in their childhood. The CTQ is based on five subscales (emotional abuse, physical abuse, sexual abuse, emotional neglect, and physical neglect), which consist of 5 items each and an additional three-item Minimization/Denial scale denoting the potential underreporting of maltreatment. Scores on the total scale of the CTQ range from 25 to 125.

#### State and trait affect

State and trait positive and negative affects were measured with the *Positive and Negative Affect Schedule* (PANAS^64^). The PANAS consists of 10 negative and 10 positive adjectives, rated on a five-point Likert scale. In the trait version of the PANAS, participants were asked to describe how they feel in general, whereas in the state version, they were asked, how they feel at the present moment.

#### Depression

We used the *Beck Depression Inventory* (BDI-II^65^) to assess the severity and presence of depressive symptoms. The BDI-II is composed of 21 items relating to symptoms of depression such as hopelessness, irritability, negative cognitions as well as physical symptoms experienced during the last 2 weeks. Every item of the BDI-II consists of four response options, which are graded by severity.

#### Anxiety

All participants completed the state and trait versions of the *State and Trait Anxiety Inventory* (STAI^66^) to measure their current and dispositional anxiety. Both versions of the STAI-20 consist of 20 items. The resulting score can vary between 20 and 80, respectively, with lower values indicating less anxiety than higher values.

#### Alexithymia

Alexithymia was assessed through the *20-Item Toronto Alexithymia Scale* (TAS-20^67^). The TAS-20 consists of three subscales: Difficulties identifying feelings, Difficulties describing feelings, and Externally oriented thinking. All items are rated on a 5-point Likert scale. The total score of the TAS-20 ranges from 20 to 100.

### Measure of intelligence

Verbal intelligence of the participants was evaluated by the *Mehrfachwahl-Wortschatz-Intelligenztest* version B (MWT-B^68^). The MWT-B includes 37 items and has no time restrictions. Each item is represented by a line, comprising four pronounceable pseudo- words (fictitious words) and one real word. It is the subject’s task to find the correct response. Subsequently, raw scores (number of items answered correctly) can be converted to IQ-scores.

### Dot-probe task

**Figure 6:**
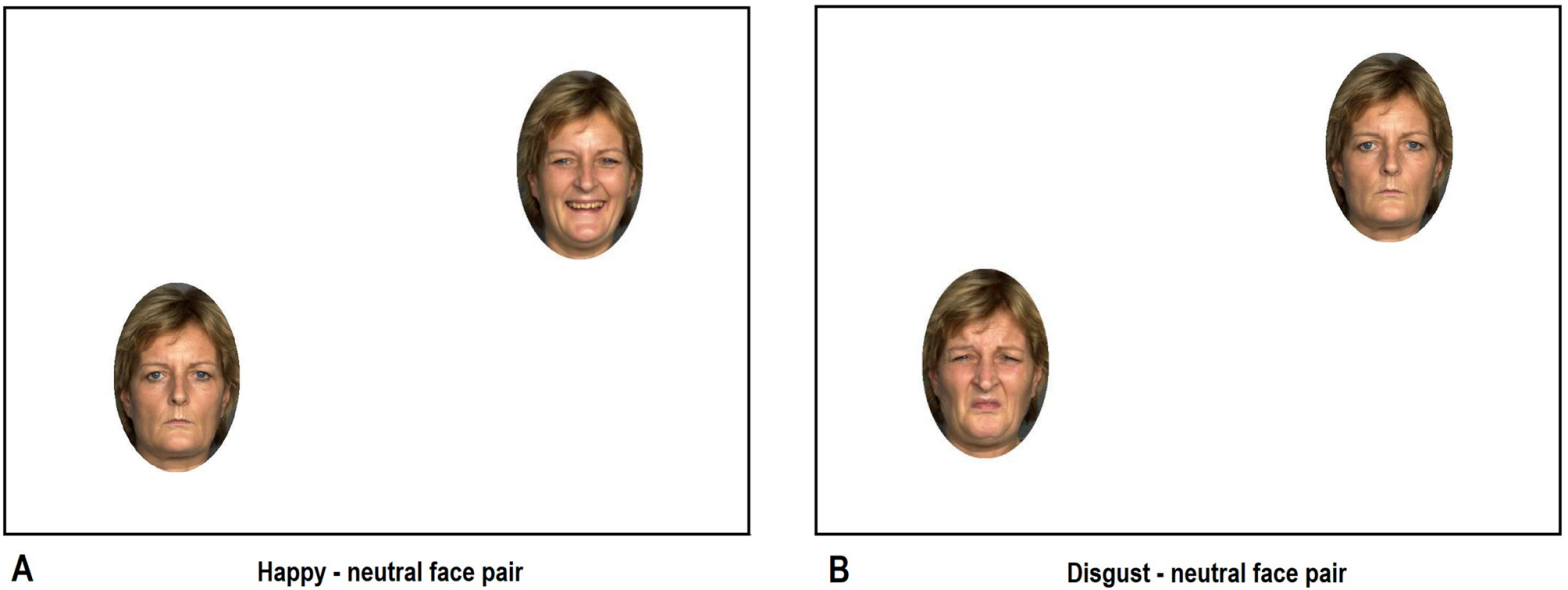
Examples of pairs of facial expressions presented in the dot-probe task.

We administered a reaction time dot-probe paradigm, to assess participants’ attention allocation to emotional facial expressions. Participants were presented with facial expression pairs, consisting of one emotional (happy, sad, or disgusted) and one neutral facial expression of the same woman. Facial expressions were taken from the FACES database^69^. Faces from 20 women were shown. 120 trials were presented in the dot-probe task (40 trials per emotion condition). Faces were brought into an oval shape to remove background information (see Fig. 6). Each trial began with the presentation of a fixation cross at the center of the screen for 500 ms. The facial expressions were presented at the I. (top right) and III. (bottom left) quadrant of the screen, with equal distance to the center (see Fig. 6). These locations were chosen to account for the possible effect of reading direction^49^. After 1000 ms the facial expressions disappeared and either a rhombus or a circle was presented at the location of the neutral or the emotional facial expression. The locations of the facial expressions and the target symbols were counterbalanced throughout the trials. Subjects were asked to respond to the target symbol as quickly as possible by pressing r if a rhombus was presented and k if a circle was presented. In a congruent trial, the symbol appeared at the location of the emotional facial expression, whereas in an incongruent trial, the target appeared at the location of the neutral facial expression. Faster responses during congruent trials than during incongruent trials indicate an attention bias towards emotional facial expressions.

### Apparatus

All stimuli were presented in their original color against a white background on a 22-inch TFT widescreen monitor (resolution: 1680 × 1050) running with an SMI-customized Dell laptop (IView X laptop). Eye movements were continuously recorded using an IView X RED250 remote system by SensoMotoric Instruments (SMI), an infrared video-based eye-tracking device sampling eye movements every 4 ms (250 Hz) with a gaze position accuracy of 0.4°. The SMI RED250 tracker is capable of compensating for changes in head position, therefore no head-resting device was required. SMIs Experiment Center software was used to present stimuli and to synchronize with recorded eye movements.

### Statistical analysis

A velocity-based algorithm with a minimum fixation duration of 100 ms, a minimum saccade duration of 22 ms and a peak velocity threshold of 40°/s was used to record the eye-tracking data. Response time in the dot probe task was defined as the latency between target onset and the participants’ key response. Eye tracking and response time data were pre-processed with BeGaze 3.0. The software was used to define two areas of interest (AOI), corresponding to the oval emotional and neutral facial expression in each trial. Statistical analyses were performed in R (version 3.6.3) using RStudio (version 1.1.383; RStudio, Inc.).

#### Correlation analyses

We used the *rmcorr* function from the *rmcorr* package (version 0.4.4) to compute the repeated-measure correlation^70^ and to explore the within-individual relationships between response times in the dot-probe task and entry times and dwell times on each facial expression type (see Table 2). An additional Product-moment correlation analysis was employed to investigate the association between self-report and test scores of the participants (see Table 1).

#### Dot-probe task

We used a linear mixed effect model, using the *lmer* function from the *lme4* package (version 1.1-27.1), to assess the effect of childhood maltreatment (CTQ score), location (bottom left, top right), congruency (congruent, incongruent) and facial expression pair (happiness, sadness, and disgust) on mean reaction time in the dot probe task. All other above-mentioned test and questionnaire scores were further integrated into the model as control variables (i.e., positive and negative state and trait affect, state and trait anxiety, depressive symptoms, alexithymia, and intelligence). Using the *anova* function from the *stats* package (version 3.6.3.), we then computed the ANOVA statistics with the Satterthwaite's method to test the effects of the independent variables on response time. Eta square effect sizes are reported for significant effects.

#### Eye tracking

We used the eye tracking parameter dwell time as indicator of late attention allocation. Dwell time relates to the duration of gaze on each facial expression type and was defined as the sum of durations (in milliseconds) of all fixations and saccades on the area of interest, i.e., the emotional or the neutral facial expression. Dwell times were computed for each facial expression type during each trial and then averaged for every participant.

The eye tracking parameter entry time was used to assess initial gaze orientation. Entry time was defined as the time (in milliseconds) between stimulus onset and the first fixation on each facial expression type. Entry times were calculated for each facial expression type and each trial and then averaged for every participant. Dwell times and entry times were analyzed using a linear mixed effect model with facial expression type (emotional, neutral), facial expression pair (happiness-, sadness-, and disgust-neutral), and CTQ score as independent variables. Again, all test and questionnaire scores were implemented in the model as control variables. Subsequently, ANOVA statistics were computed to test the effects of the independent variables on entry and dwell time. If indicated, post-hoc pairwise comparisons by the *emtrends* function of the *CRAN* package (version 1.7.0) were used to compute and test the estimated marginal slopes. Eta square effect sizes are presented for significant effects.
